# Real-time quantitative monitoring of hiPSC-based model of macular degeneration on Electric Cell-substrate Impedance Sensing microelectrodes

**DOI:** 10.1016/j.bios.2015.04.079

**Published:** 2015-09-15

**Authors:** W. Gamal, S. Borooah, S. Smith, I. Underwood, V. Srsen, S. Chandran, P.O. Bagnaninchi, B. Dhillon

**Affiliations:** a MRC Centre for Regenerative Medicine, The University of Edinburgh, EH16 4UU, United Kingdom; b Institute for Bioengineering, School of Engineering, The University of Edinburgh, EH9 3DW, United Kingdom; c Institute for Integrated Micro and Nano Systems, School of Engineering, The University of Edinburgh, EH9 3JF, United Kingdom; d Centre for Clinical Brain Sciences, The University of Edinburgh, EH16 4SB, United Kingdom; e Euan MacDonald Centre for MND Research, The University of Edinburgh, EH16 4SB, United Kingdom; f Centre for Neuroregeneration, The University of Edinburgh, EH16 4SB, United Kingdom; g The Anne Rowling Regenerative Neurology Clinic, The University of Edinburgh, EH16 4SB, United Kingdom; h School of Clinical Sciences, The University of Edinburgh, EH16 4SB, United Kingdom

**Keywords:** Tissue-on-a-chip, Impedance sensing, Wound healing, Human induced pluripotent stem cells, Macular degeneration, Disease model

## Abstract

Age-related macular degeneration (AMD) is the leading cause of blindness in the developed world. Humanized disease models are required to develop new therapies for currently incurable forms of AMD.

In this work, a tissue-on-a-chip approach was developed through combining human induced pluripotent stem cells, Electric Cell–substrate Impedance Sensing (ECIS) and reproducible electrical wounding assays to model and quantitatively study AMD. Retinal Pigment Epithelium (RPE) cells generated from a patient with an inherited macular degeneration and from an unaffected sibling were used to test the model platform on which a reproducible electrical wounding assay was conducted to model RPE damage. First, a robust and reproducible real-time quantitative monitoring over a 25-day period demonstrated the establishment and maturation of RPE layers on the microelectrode arrays. A spatially controlled RPE layer damage that mimicked cell loss in AMD disease was then initiated. Post recovery, significant differences (*P*<0.01) in migration rates were found between case (8.6±0.46 μm/h) and control cell lines (10.69±0.21 μm/h). Quantitative data analysis suggested this was achieved due to lower cell–substrate adhesion in the control cell line. The ECIS cell–substrate adhesion parameter (*α*) was found to be 7.8±0.28 Ω^1/2^ cm for the case cell line and 6.5±0.15 Ω^1/2^ cm for the control. These findings were confirmed using cell adhesion biochemical assays. The developed disease model-on-a-chip is a powerful platform for translational studies with considerable potential to investigate novel therapies by enabling real-time, quantitative and reproducible patient-specific RPE cell repair studies.

## Highlights

• hiPSC-RPE model was established and characterised on ECIS microelectrode arrays.
• A reproducible electrical wound healing assay was used to mimic RPE cell damage.
• Significant differences in migration rates were found between case and control.
• Cells recovered through mesenchymal migration mirrored by impedance fluctuations.
• Case cell line showed significantly higher adhesion than the control cell line.

## 1 Introduction

Age-related Macular Degeneration (AMD) is the most common cause of blindness in the developed world (Johnson et al., 2005).The disease usually affects patients in their seventh and eighth decades resulting in the loss of functionally important central vision. There are two main forms of the disease; wet AMD, in which sub-retinal vascular leakage predominates and dry AMD in which deposit formation and cell degeneration are the primary disease processes. Although treatments have recently been developed for wet AMD there is currently no effective treatment for dry AMD. The Retinal Pigment Epithelium (RPE) is a pigmented, polygonal monolayer of cells found directly below the photoreceptor layer in the retina. The primary role of the RPE is in photoreceptor homeostasis. RPE dysfunction, degeneration and reduced repair are implicated in the AMD disease process (Cai and Del Priore, 2006; Thurman et al., 2009). In normal ageing, RPE cell loss is compensated for by increasing cell size and migration of neighboring RPE (Panda-Jonas and Jonas, 1996). In AMD however, healing is hampered leading to problems such as incomplete RPE coverage and the consequent death of overlying photoreceptors. Late onset retinal macular degeneration (LORMD) is a rare, autosomal dominant form of macular degeneration (Hayward et al., 2003). LORMD shares key clinical and pathological features with AMD including dark adaptation delay, drusenoid retinal changes prior to sub-retinal deposit, RPE cell loss and neuro-retinal atrophy and has thus been proposed as a good model for AMD (Borooah et al., 2009). In vitro RPE models are required to investigate the underlying mechanisms associated with AMD. However, it is difficult to obtain and culture primary functional human retinal cells, hence the predominance of immortalized cell lines in AMD research (Borooah et al., 2013). In most cases though, the behavior of these immortalized lines poorly reflects what actually happens in vivo (Alge et al., 2006; Klimanskaya et al., 2004. Human induced Pluripotent Stem Cells (hiPSCs) technology offers a novel approach for disease modeling, with the potential to impact translational retinal research and therapy. Recent developments enable the generation of RPE cells derived from patients (hiPSC-RPE) thus allowing in vitro study of human retinal disease that has greater clinical and physiological relevance (Meyer et al., 2009; Takahashi et al., 2007). In this study, RPE was derived from both a patient with an LORMD and from an unaffected sibling (Borooah et al., 2009).

In addition to the limitations of in vitro RPE models, a number of issues associated with the study of wound healing in biology have hampered the development of relevant AMD models. Mechanical wounding methods including scratching (Li et al., 2009; Fronzaa et al., 2009) and stamping are the most commonly used wound healing assays (Lee et al., 2010). Scratching assays however does not produce a highly repeatable wound; a disadvantage that can be overcome by stamping. These methods can also disrupt the ECM layer as well as form debris that might affect the migration process. Gap closure assays can be used to produce cell free regions where cell migration can be monitored after removing a solid (Poujade et al., 2007), liquid (Doran et al., 2009) or gel barrier (Varani et al., 1978). Cells grow around the physical barrier and migrate upon removal. Other wounding techniques include chemical (Legrand et al., 1999) and optical methods (Zordan et al., 2011). However, most of the above methods require extensive manipulation of the cell layer during both wounding as well as repair processes. Moreover, problems of quantification and reproducibility occur due to the difficulty of controlling the wounded areas.

In order to overcome both cell source and current wounding assays limitations, a tissue-on-a-chip approach was investigated by developing and characterising a human induced pluripotent stem cells model of RPE layer on Electric Cell–substrate Impedance Sensing microelectrode arrays. Electric Cell–substrate Impedance Sensing (ECIS) is a technology pioneered by Giaever and Keese in which a small non-invasive AC current (1 μΑ) is applied using gold microelectrodes (Giaever and Keese, 1984; Giaever and Keese, 1991). Changes in the time-course complex impedance associated with cellular events are monitored by means of in-phase and out-of-phase measurements using a lock-in-amplifier (Giaever and Keese, 1991). ECIS has found many applications from cell attachment and spreading (Wegener et al., 2000; Lo et al., 1995), signal transduction (Han et al., 2009), cytotoxicity (Opp et al., 2009; Lovelady et al., 2009) to metastasis (Keese et al., 2002) and more recently regenerative medicine (Bagnaninchi and Drummond, 2011). ECIS migration assays also allow the combination of electrical wounding and impedance spectroscopy to quantitatively and reproducibly measure cell migration rates and changes in cell morphology accompanying wound healing (Wegener et al., 2000).

In this paper, the need for a physiologically relevant in vitro model of RPE layer repair that can be investigated quantitatively and reproducibly has been addressed through developing a tissue-on-a-chip approach. First the hiPSC-RPE model was established and characterised on ECIS microelectrode arrays. Then an electrical wound healing assay was used to mimic RPE cell damage in both control and diseased hiPSC-RPE cell lines to study differences in repair. Finally the disease model-on-a-chip was used to answer a series of questions concerning the role of RPE adhesion in repair pointing towards potential therapeutic strategies.

## 2 Materials and methods

### 2.1 Generation of hiPSC-RPE lines

Derivation of hiPSC lines from one patient with late-onset retinal macular degeneration (LORMD) and one unaffected sibling was achieved using previously established methods (Bilican et al., 2012; Yusa et al., 2011). RPE differentiation was then established using a variation of a previously published protocol (Meyer et al., 2009). For more details on the protocols used, see Supplementary data. The cells were maintained as adherent cultures in retinal differentiation medium (RDM) until the appearance of pigmented RPE cells. Large patches of pigmented RPE cells were micro-dissected and then grown on laminin coated plates initially in 10% FBS/RDM 90% for two days, followed by 2% FBS/98% RDM till confluent before switching to RDM. RPE validation was performed using RT-PCR and immunostaining.

### 2.2 Quantitative real-time PCR (RT PCR)

Total RNA was extracted using the RNAeasy Mini Plus Kit (Qiagen) and treated with DNase1 to remove any genomic DNA contamination. Synthesization of cDNA was performed using a cDNASynthesis Kit (Thermo Scientific), and PCR (34 cycles) was performed using gene-specific primers. PCR products were analyzed on 2% agarose gels. Quantitative RT PCR experiments (40 cycles) were carried out using SYBR® Green Supermix (Bio-Rad) and a Bio-Rad C1000 thermal cycler, and results were analyzed using Bio-Rad CFX software and Microsoft Excel.

### 2.3 Tissue culture on microelectrode arrays

Case and Control hiPSC-RPE were cultured on ECIS medusa arrays (Applied Biophysics, NY, USA) incorporating a 2-electrode set up: working and counter electrodes. Each array has 8 wells of 0.8 cm^2^ surface area with two 250 μm diameter gold working microelectrodes per well. Microelectrodes are fabricated on a transparent Lexan polycarbonate substrate onto which 50 nm thick gold electrodes are sputtered and passivated with 2 µm polymer resin. Sensing electrodes have a typical area of 0.05 mm^2^ while the counter electrode has an area of 18 mm^2^. The spacing between the electrodes on a medusa array is 4.83 mm. Because the area of the counter electrode is much larger than that of the working electrode, its impedance contribution can be neglected. The two working electrodes are addressed individually and measurements from one working electrode are recorded at a time. The ECIS electrode array was placed in an array holder inside a humidified incubator at 37 °C and 5% CO_2_. All wells were incubated for 2 h with the culture medium before seeding at confluency (100,000 cells/well). Cells were initially cultured with retinal differentiation medium with 10% fetal calf serum. On day 2 of culture, the media was changed into RDM 2% serum and on day 6, RDM with no serum was used until the end of the experiment. Media was changed 3 times per week. Cells were cultured for 25 days to achieve RPE maturation before starting the wound healing assay.

Immortalized RPE cell lines with hTERT (hTERT-RPE1, ATCC) were cultured in DMEM-F12 (ATCC) with 10% fetal bovine serum (FBS) and 0.01 mg/ml hygromycin B. Cells were seeded with a density of 40,000 cells/well and cultured for two days before starting the migration assay. Culturing medium was changed 3 times per week.

### 2.4 Impedance sensing

A commercial Electric Cell–Substrate Impedance Sensing instrument (ECIS Z⦵, Applied Biophysics) was used to record multiple frequency impedance measurements (62.5, 125, 250, 500, 1000, 2000, 4000, 8000, 16,000, 32,000 and 64,000 Hz) every 160 s. The measured complex impedance had a resistive and capacitive component which was determined through in-phase and out-of-phase measurements using a lock-in amplifier.

In this study spectroscopic impedance data were presented only at low (4 kHz) and high frequency (64 kHz) for clarity reasons. Following the work of Wegener et al., the resistance at 4 kHz is presented and reflects a combination of intercellular (establishment of cell–cell junctions) and subcellular (cell–substrate adhesion) alterations as well as cell motility (Wegener et al., 2000). In contrast, the capacitance measurements at 64 kHz translate linearly in cell coverage as most of the current is intracellular at this frequency (Wegener et al., 2000).

### 2.5 Wound healing assay

Generally, impedance sensing with ECIS is achieved with a non-invasive current of 1 µA generated by 1 V, AC signal passing through a 1 MΩ resistor. However, a higher AC voltage (from 100 mV to 3 V) can be applied through a 1 kΩ resistor, thus generating an elevated current (0.1 mA to 3 mA), to wound the cell layer. In this study, an elevated current pulse (3 mA, 40 kHz, 30 s) generated by the ECIS microelectrodes and optimized by trial-and-error experiments was used to wound the confluent cell monolayer. The wounding pulse was mirrored by a drop in impedance to that of the cell free electrode (Fig. 2(a)). The system then switched back to its normal operation to monitor cell repair (Keese et al., 2004). The same wounding parameters were used with all cell lines.

In some cases, trypan blue was used to confirm cell death. 15 min after wounding, the medium was aspirated and 200 μl of diluted trypan blue was added to the well under investigation. After 15 min, trypan blue was removed and the wounded cells were optically observed. Only dead cells were stained blue as their plasma membranes were terminally compromised.

### 2.6 Quantitative data analysis

#### 2.6.1 Healing kinetics

ECIS data was exported to Matlab for further analysis. The mean and standard errors were calculated for the different cell lines. To better understand the healing kinetics, the healing graphs were fitted to a sigmoid curve in which the hill slope and inflection points were calculated using Eq. (1) shown in Supplementary data, thus providing additional data on the differences between case and control migration.

#### 2.6.2 Average cell migration

The migration rate of the case and control cell lines after electrical wounding was determined according to Eq. (2) in Supplementary data.

#### 2.6.3 Impedance based adhesion assay

**Fig. 1:**
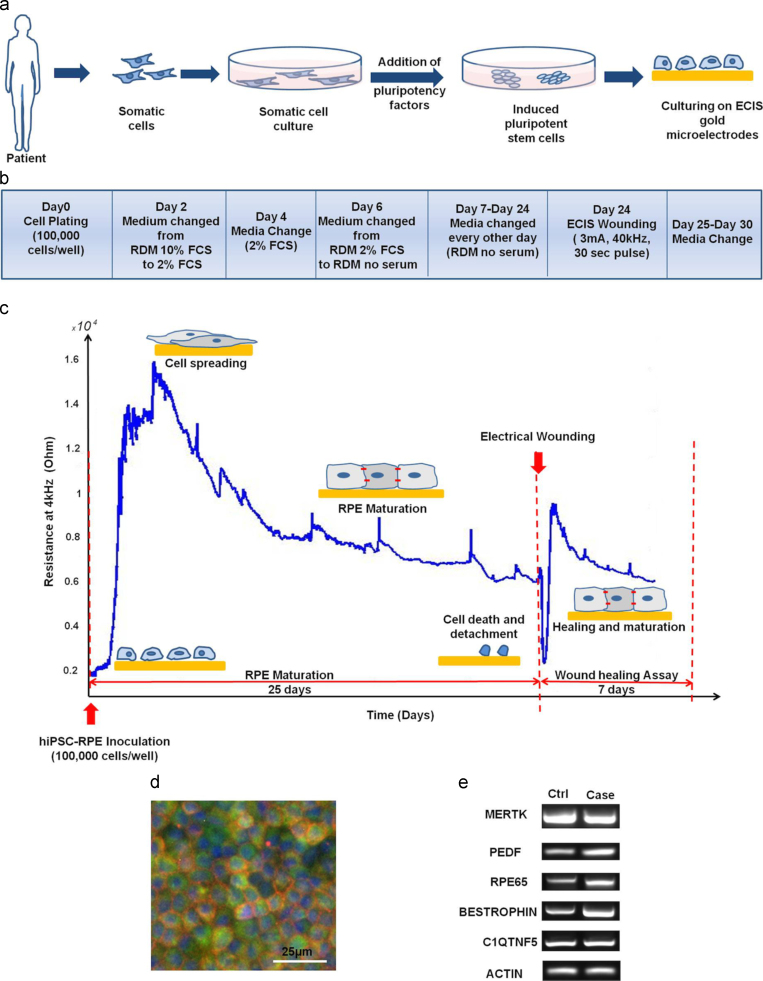
Development and characterization of the hiPSC-RPE model on ECIS microelectrodes. (a) Patient's fibroblasts were expanded and reprogrammed with Yamanaka factors Klf4, Oct3/4, Sox2, c-Myc to a pluripotent state before being differentiated to RPE. Cells were plated (Day 0) on ECIS microarrays before being allowed to mature for an additional 3 weeks period. (b) Timeline protocol: Cells were cultured with a density of 100,000 cells/cm^2^. Culturing medium was switched from RDM 10% FCS to RDM 2% FCS on Day 2 of culture, and then to RDM no serum on Day 6. Medium was changed every other day for 25 days until RPE maturation was obtained. (c) The complex impedance was monitored in real-time throughout RPE maturation and is displayed as the resistance at 4 kHz. It showed an increase with cell spreading and maturation followed by a decrease reflecting changes in cell morphology and size. After 25 days, an automated electrical wound healing assay was performed and subsequent cell migration associated with healing phase was monitored. (d) Immunostaining showing the expression of the transmembrane RPE specific protein Bestrophin(red), Ezrin (green) and DAPI (blue) illustrated markers of mature RPE. (e) RT-PCR revealed that both case and control hiPSC-RPE lines expressed global epithelial and RPE specific markers.

Cells were seeded with a high density (typically 100,000 cells/cm^2^) and the changes in their impedances were monitored for 1–2 days. The time it takes the cells to attach to the electrode surface and start spreading till they form a confluent layer is an indicator of the cell adhesion properties (Wegener et al., 2000; Heijink et al., 2010) (Fig. 1(d)). Attachment kinetics was fitted to a linear fit and the slope of the case and control curves were determined accordingly. A biochemical adhesion assay was also performed to confirm these results (See Supplementary data).

#### 2.6.4 Cell–substrate adhesion parameter

The built-in ECIS model (Giaever and Keese, 1991) was used to retrieve the cell adhesion parameter (*α*) to investigate the differences in cell–substrate adhesion between the cell lines under investigation. This model assumes cells as circular disks with radius (*r*) hovering above the electrode at a distance (*h*) in a culture medium with resistivity (*ρ*). The different current pathways (between, under and tough the cells) are then analyzed using differential equations to derive a transfer function defining the complex impedance and the model parameters. The ECIS adhesion parameter (*α*) is derived according to Eq. (1) and is a reflection of how close the cells are to the electrode. A small cell–electrode distance (*h*) indicates higher cell–electrode adhesion, i.e. more resistivity against the current flow underneath the cells.

(1) $$\alpha =r\sqrt{\frac{\rho }{h}}\;\;(\Omega^{1/2}\mathrm{cm})$$

A schematic diagram of the ECIS model is shown in Supplementary Fig. 1(c).

#### 2.6.5 Moving variance

Matlab was used to analyze the increase in monitored impedance fluctuations accompanying cell differentiation by the moving variance method (Schneider et al., 2011). First the signal was detrended and normalized. Then the variance was calculated over a total of 512 points (22.75 h) within a 150 point window and a 1 point sliding step, and plotted against time.

#### 2.6.6 Statisitcs

Matlab was also used to perform one-way Anova and Tukey–Kramer multicomparison tests to determine whether the groups under investigation were significantly different from each other. A probability value of *P*<0.01 was set as significant.

## 3 Results

### 3.1 An hiPSC-RPE model on ECIS microelectrode arrays

Induced pluripotent cells were successfully derived from a patient with late-onset retinal macular degeneration (LORMD) and one unaffected sibling. In each case the cells were then differentiated towards RPE cells before being plated on ECIS microelectrode arrays (Fig. 1(a)). Final RPE maturation was completed on the microelectrode arrays using a stepped 25-day protocol that progressively moved towards a serum-free medium (Fig. 1(b)). The hiPSC-RPE control and case cell lines were plated at confluency onto ECIS medusa arrays (Supplementary Fig. 1(a)) with a density of 100,000 cells/well on day 0. Retinal differentiation medium (RDM) was used as the culturing medium with the concentration of fetal bovine serum being changed from 10% to 2% on day 2 and then to 0% from day 6 onwards. Maturation of RPE took place on top of the gold microelectrodes and was evidenced through the changes in ECIS measurements.

Real-time quantitative monitoring of the spectroscopic complex impedance, with a resistive and capacitive component, was performed throughout the RPE maturation phase by acquiring multi-frequency data points at a 160 s interval. The 25-day time-course maturation process was found to be highly robust and reproducible for both case (*N*=9) and control wells (*N*=11) thus offering a criterion by which to reject faulty in vitro models. Fig. 1(c) shows the changes in the resistance measured at 4 kHz that characterised RPE maturation and was associated with initial cell spreading, morphological changes and barrier formation with an average standard error of 144.6 Ω for the control cell line and 203.2 Ω for the case. The resistance increased with cells attaching and spreading onto the electrodes. First a steep increase in resistance that peaked at 15 kΩ was observed on day 2 of culture and this was followed by a slow decrease over 5.5 days to a plateau with an average value of 8.5 kΩ. The formation of the confluent cell layer and the hiPSC-RPE differentiation stages were also observed under a microscope. It was noticed that the drop in ECIS measurements following the peak in resistance was mirrored by a change in cell morphology from fibroblastic-like cells towards polygonal cobble-stone mature epithelial cells.

Finally, Fig. 1(c) shows that after RPE maturation, an electrical wound healing assay was conducted directly on the chip as schematically described in Supplementary data (Supplementary Fig. 1(b)). An electrical pulse (3 mA, 40 kHz, 30 s) was used to injure the cells on top of the electrode creating a reproducible 250 μm circular wound that mimicked cell loss and damage associated with macular degeneration. A drop in resistance to the level of the cell-free electrode (2600 Ω) was monitored followed by a gradual increase reflecting the migration of RPE cells to repopulate the microelectrodes after wounding.

Before conducting the wound healing assay, the establishment and maturation of the RPE layer on top of the microelectrodes were confirmed. Immunostaining (Fig. 1(d)) and RT-PCR analysis (Fig. 1(e)) were used to identify RPE markers indicating RPE maturation.

RT-PCR showed that both cell lines expressed the global epithelial and RPE specific markers including the phagocytosis marker MERTK, the basal marker BEST1, the apical membrane associated marker Ezrin, the pigmentation marker PEDF(pigment epithelium derived factor), the visual cycle marker RPE65 as well as C1QTNF5 and ACTIN. Moreover, qPCR was used to analyze the relative fold change of expression of the selected mRNA of RPE relative to iPS (Supplementary Fig. 2).

Taken together these data show that an hiPSCs-derived model of the RPE layer can be developed directly on ECIS gold microelectrode arrays and that its robustness, reproducibility and suitability can be quantitatively addressed by real-time impedance sensing before initiating an integrated electrical wound healing assay.

### 3.2 Electrical wound healing assays of the hiPSC-RPE layer

After 25 days, a mature RPE layer that entirely covered the bottom of the culture well including the gold microelectrodes was established. An RPE injury (analogous to focal RPE loss observed in macular degeneration) was then created as a circular wound with an elevated electrical pulse in the microelectrode area. Then the wound healing process was monitored for both case and control cell lines with ECIS.

**Fig. 2:**
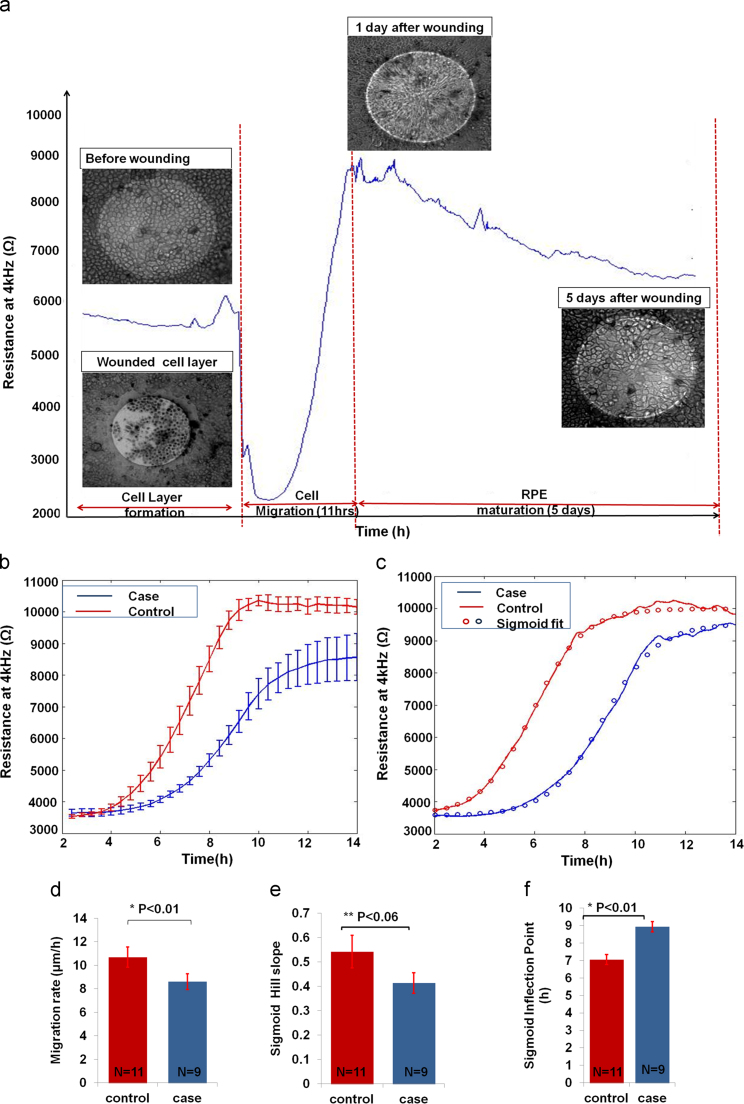
ECIS Wound Healing Assay of hiPSC-derived RPE. (a) Wounding hiPSC-RPE: The resistance kinetics for one case study after wounding and during healing was monitored. Cells were wounded using wounding parameters of 3 mA, 40 kHz, 30 s. Trypan blue was used to stain dead cells that did not detach from the electrodes. (b) Wounding was a reproducible process, producing a defined wound every time. The control cell line migrated faster than the case cell line to achieve wound healing. (c) Sigmoid fitting: the case and control healing curves were fitted to a sigmoid curve. The parameters of the fitting curve were further analyzed. (d) Migration rate bar graphs: the control migration rate was 10.69±0.21 μm/h while that of the case cell line was 8.6±0.46 μm/h. The two rates were significantly different (*P*=0.0046). (e) Sigmoid hill slope: the difference between the control and case hill slopes were not significantly different. (f) Inflection points were found significantly different.

Electrical pulse parameters have been optimized through trial and error experiments. A 3 mA current pulse at 40 kHz applied for 30 s was found to cause cell death while preserving the microelectrode integrity. Fig. 2(a) shows the corresponding drop in R_4kHz_ that followed the electrical wound. R_4kHz_ reached a value of 2600 Ω corresponding to a cell-free electrode. This indicated cell death. Light microscopy confirmed that some cells detached from the microelectrode while the few remaining were stained positively with trypan blue indicating irreversible electroporation and death.

Wound healing was then monitored until the RPE layer fully recovered (5 days), and was characterised by two distinct phases (Fig. 2(a)). First, the cells from the wound edges migrated underneath the layer of dead cells in a radial pattern to close the wound. They repopulated the microelectrode with a characteristic steep increase in resistance followed by a plateau that indicated the end of cell migration. Then a maturation phase was observed where the resistance slowly decreased to a second plateau in a similar way to that described for early RPE maturation in Fig. 1(c). Light microscopy indicated that once cell migration was complete, elongated cells switched back to a cuboidal morphology indicating RPE maturation at the end of this phase.

The mean and standard error of the migration phase for all case and control cell lines is shown in Fig. 2(b). It shows that wounding was a reproducible process, producing a defined and concise wound every time. The corresponding capacitance measurements at 64 kHz are shown in Supplementary Fig. 3(a).When calculating the migration rate as the time to repopulate a 250 μm electrode (Fig. 2(d)), we found that the control cell line had a significantly (*P*=0.0046) higher migration rate (10.69±0.21 μm/h) than the case cell line (8.6±0.46 μm/h).

In order to gain more insight into the healing kinetics, R_4kHz_ was fitted to a sigmoid along the migration phase as exemplified for one case and one control cell line in Fig. 2(c). No significant difference (*P*=0.057) was found between the hill slope as shown in Fig. 2(e) of the control (0.54±0.07) and that of the case cell lines (0.41±0.04). However, there was a significant difference between the case and control at the sigmoid inflection point. Fig. 2(f) shows that the inflection point had a significantly (*P*=0.0009) higher value for the case cell line (8.94±0.29 h) than that of the control cell line (7.06±0.27 h).

These results suggested that the case lower migration rate (i.e. the longer time taken by the cells to repopulate the electrodes) of the case cell line might be attributed to a delay in initiating migration. Once the cells rearranged themselves radially and started to move, they migrated with a speed similar to that of the control cell line.

### 3.3 Observed similarities between healing and maturation processes

**Fig. 3:**
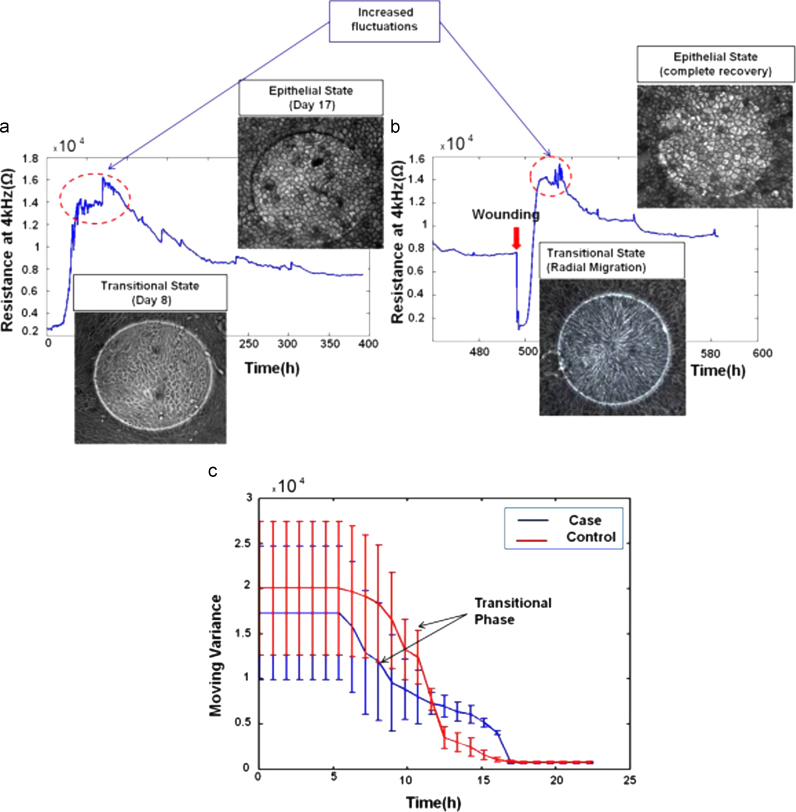
Similarities between early RPE maturation and healing processes. (a) Differentiation and morphology changes: case and control cell lines changed their morphology from a fibroblastic-like to a cuboidal shape during the differentiation and maturation stage. This was accompanied by an increase in measurement fluctuations. The resistance decreased when the epithelial stage was reached which was attributed to the change in cell size and morphology. (b) Wounding and morphology changes: unwounded cells changed morphology from cuboidal to elongated in order to migrate and repopulate the electrodes. The reverse process then occurred after closing the wound. (c) Moving Variance showed a clear transition phase associated with a transition from an elongated to a cuboidal morphology.

During the wound healing process, a transitional stage was observed in which cells showed a change in morphology which was similar to the changes accompanying the early RPE maturation although on a much shorter time scale (Fig. 3(a)). Cells around the wound edge went through a transition from cuboidal to elongated cells before returning to their original polygonal morphology after repopulating the electrodes (Fig. 3(b)). Increased fluctuations were also observed for these transitional phases. A moving variance analysis was applied to the ECIS measurements after wounding following the work of Schneider et al. (2011) (Fig. 3(c)). The analysis clearly shows the transition from a high cellular activity (high variance phase) to a more quiescent state (low variance). No significant differences were found between the case and control cells with an average half-time transition of 11.5 h.

These data suggested that the case cell’s ability to go back to the original RPE morphology after completing migration was not affected by the LORMD mutation. Cell morphology and impedance data strongly suggested that cells were able to close the wound through integrin-mediated “mesenchymal” migration (Huttenlocher and Horwitz, 2011).

### 3.4 Cell–substrate adhesion properties

One of the advantages of the tissue-on-a-chip approach is the ability to exploit the quantitative impedance data collected throughout RPE maturation and wound healing.

**Fig. 4:**
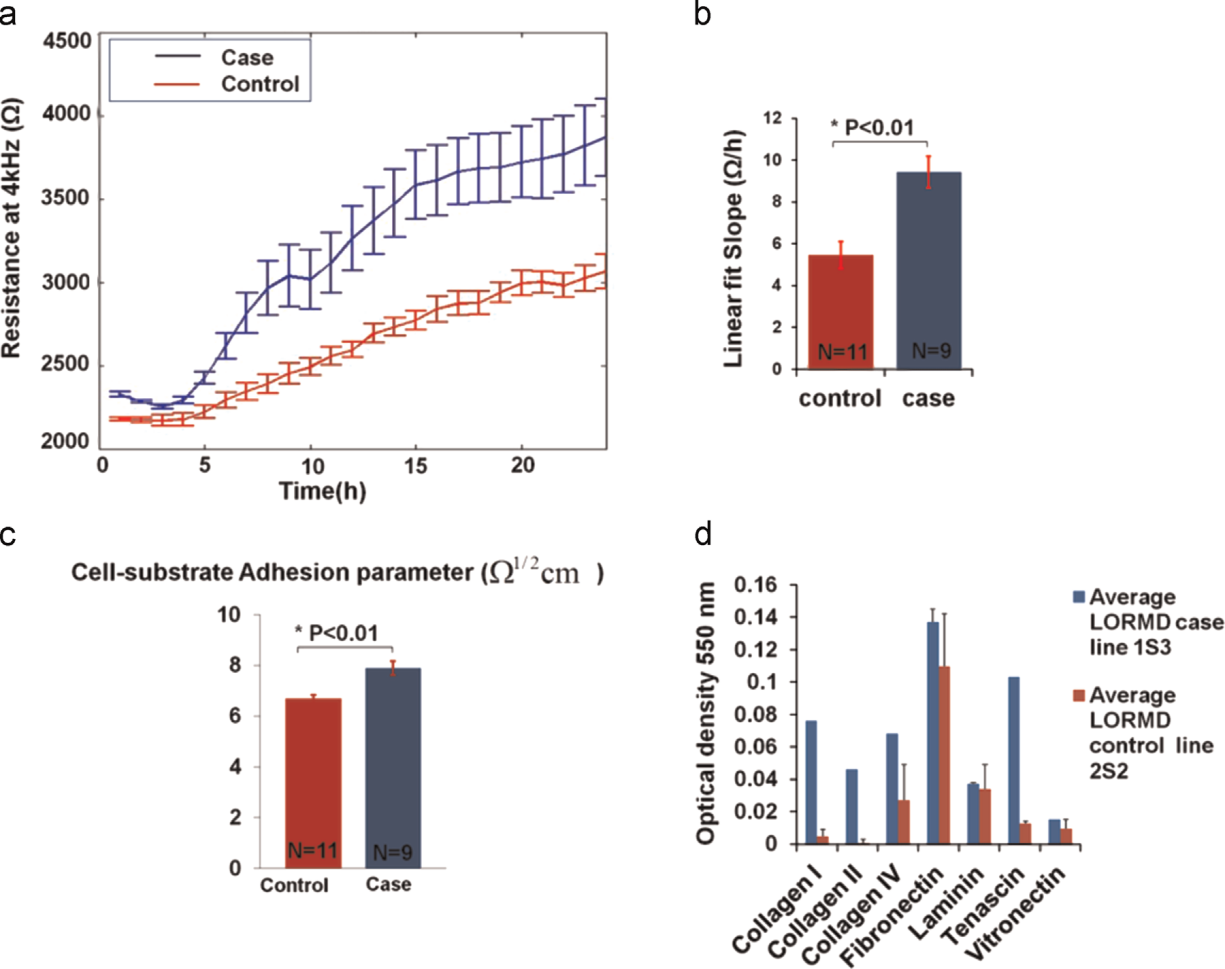
Case vs. control cell–substrate adhesion. (a) The hiPSC-RPE ECIS adhesion assay: comparing the resistances of the case and control cell lines during the first 24 h of culture, showed how the case cell line attached more quickly to the electrodes and were able to reach a plateau faster than the control cell line. (b) Attachment slope: the slopes of the attachment curves for both case and control cell lines were calculated through a linear fit. The slope was significantly higher for the case cell line (*P*<0.01) which was consistent with the case cells arriving at a plateau before the control cells. (c) Cell–substrate adhesion parameter (*α*): ECIS parameter (alpha) of the case cell line was significantly higher than that of the control cell line (*P*=0.002), indicating that the case cell line obtained a higher cell–substrate adhesion. (d) Adhesion biochemical assay: the case cell line showed stronger adhesion to various ECM proteins than the control cell line.

The first monitored difference between the control and case cell lines was at the ECIS attachment and spreading phase during the first 24 h after culture. R_4kHz_ measurements showed that the case cell line attached to the electrodes and spread to form a confluent layer more readily than the control cell line (Fig. 4(a)). The corresponding capacitance measurements during the same period are shown in Supplementary Fig. 3(b).

As the case and control cell lines were seeded at confluency on the microelectrode arrays, the first 24 h of measurements were considered to constitute an impedance-based adhesion assay revealing data on the cell–substrate adhesion. Generally in these types of assays, the microelectrodes are entirely covered with a tight layer of cells that upon attachment to the electrode surface causes a significant increase in the measured resistance. The difference in the measurements between different cell lines can therefore be attributed to cell attachment and adhesion to the substrate (Wegener et al., 2000).

For further analysis, the slope of each of the attachment curves was fitted to a linear model and they were found to be significantly different (*P*<0.01) with a value of 5.45±0.64 Ω/h for the control and 9.43±0.76 Ω/h for the case cell line (Fig. 4(b)). After this attachment phase however, the case and control cell lines followed similar kinetics throughout the differentiation and maturation phases (see Supplementary Fig 4).

To further investigate the potential role of cell adhesion, the ECIS model was used to define the cell–substrate adhesion parameter (*α*) (Fig. 4(c)). Again the case cell line showed significantly (*P*<0.01) higher adhesion (7.8±0.28 Ω^1/2^ cm) than the control cell line (6.5±0.15 Ω^1/2^  cm).

These findings were confirmed by a biochemical adhesion assay (Fig. 4(d)) that showed a clear difference between the adhesion properties of the case and control cell lines. The case line showed a significantly higher adhesion to ECM proteins (including collagen IV and tenascin) than the control cell line. Note that for the case and control cells cultured on ECIS arrays, the microelectrodes were coated with a mixture of proteins present in serum prior to culture.

**Fig.5:**
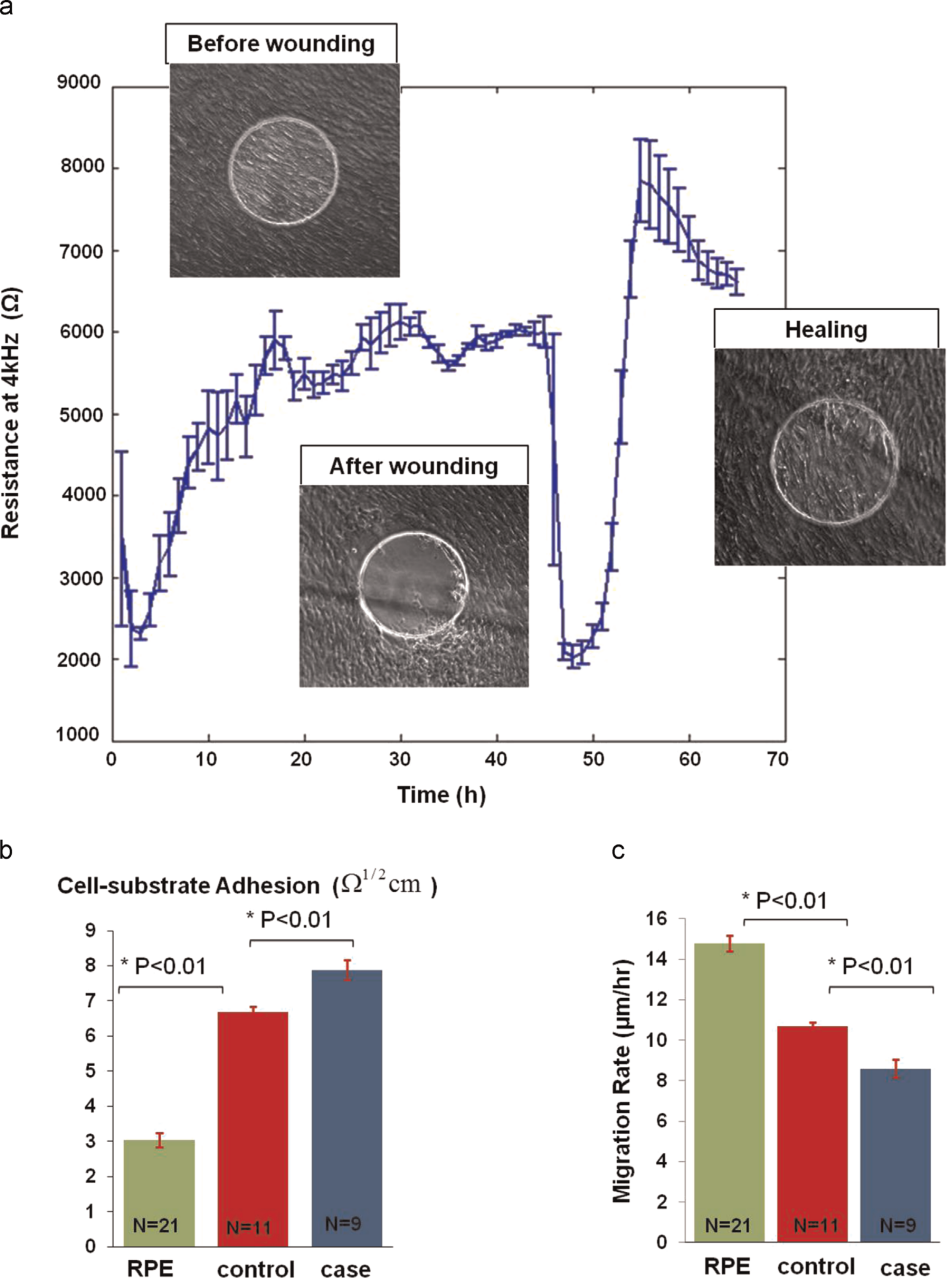
Effect of cell–substrate adhesion on the migration of immortalized and hiPSC-derived RPE (a) Wounding htert-RPE1: wounding parameters used for the migration assay were 3 mA, 40 kHz, 30 s. Wounded cells completely detached from the electrode surface upon wounding leaving clear electrodes. Wound healing was achieved as cells migrated to repopulate the electrode. (b) Cell–substrate adhesion: htert-RPE1 had the lowest adhesion parameter (*α*), followed by the control cell line and the case line with the highest *α*. (c) Migration rates: htert-RPE1 had the highest migration rate. It was followed by the control hiPSC-RPE cell line and then by the case cell line.

Finally, to explore further the link between cell adhesion and cell migration, electrical wounding of an additional RPE cell line was conducted (Fig. 5(a)). The immortalized cell line htert-RPE1 were chosen for this study as they are regularly used for in vitro RPE migration assays even with their reported limitations in reflecting in vivo behavior (Alge et al., 2006; Klimanskaya et al., 2004). After htert-RPE1 reached confluency, an ECIS wound healing assay was conducted and its healing rate and adhesion properties were compared to that of the hiPSC-RPE (Fig. 5(b) and (c)). The wounding parameters for htert-RPE1 were the same as that for the hiPSC-RPE case and control cell lines (3 mA, 40 kHz, 30 s). In contrast to the hiPSC-RPE, the wounded cells completely detached leaving a clear electrode, which already suggested that the cells adhered less to the electrodes. Comparing the cell–substrate ECIS parameters of the different cell lines showed that htert-RPE1 were the least adherent to the electrode's surface followed by the control hiPSC-RPE and then by the case cell line having the highest *α*. This was in turn reflected in the migration rates with the immortalized htert-RPE1 having the highest migration rate of 14.79±0.39 μm/h followed by the control cell line (10.69±0.21 μm/h) and then by the case line (8.6±0.46 μm/h) with the slowest migration rate of the three cell lines. These data strongly suggest that the difference observed in cell migration rate between the case and the control cell line may be attributed to a difference in cell adhesion properties.

## 4 Discussion

In this study, a tissue-on-a chip approach was developed to investigate RPE layer damage and repair in order to mimic retinal macular degeneration. Differences in wound healing between a case and control RPE cell lines associated with an inherited macular degeneration were reproducibly and quantitatively identified. The obtained results demonstrated that the healing rate was reduced in the case cell line. The differences in healing resulted from a reduced migration rate in the case RPE line when compared to the control RPE line. In addition, the cell lines were found to have different cell–substrate adhesion properties, which may be the reason behind the different cell migration rates. Moreover, the cells undergoing migration adopted a radial pattern of wound healing around the site of damage suggesting a single cell “mesenchymal” migration as opposed to collective “Ameboid” migration. (Huttenlocher and Horwitz, 2011). “Mesenchymal” migration is integrin dependent and therefore directly linked to cell adhesion properties.

Increased fluctuations in impedance measurements after wounding as well as during maturation were associated with a change in cell morphology from cuboidal to elongated shape. Increased fluctuations monitored by ECIS were reported previously by Schneider et al. (Schneider et al., 2011) and were attributed to the ruffling of cell membranes during EMT (epithelial-to-mesenchymal-transition) process. Taken together, these results point towards an EMT/MET transition post wounding which is known to play a role in development as well as migration in other cell lines (Lane and Jiang, 2012; Lane and Jiang, 2013) and in RPE (Chen et al., 2014). Both the control and case lines went through a morphological change towards a mesenchymal like state during migration before going back to a cuboidal morphology after repopulating the microelectrodes.

Although the central role of cell adhesion in cell migration is established, the relation between migration rate and adhesion level is more subtle. Generally a cell line will have an optimum cell migration rate at intermediate levels of adhesion to allow both an efficient cell–substrate attachment and release (Gupton and Waterman-Storer, 2006). In the presented study, the lower migration rate of the case cell line was found to be associated with a stronger cell–substrate adhesion than that of the control cell line. In this study adhesion and migration parameters were measured separately and thus represent independent, quantitative parameters that will help to achieve a better understanding of the complex relationship between adhesion and migration, and could be used to identify potential therapeutic agents that promote cell migration by modulating cell adhesion (lower or higher).

The case cell line (late onset retinal macular degeneration) results from a mutation in the gene encoding the protein C1QTNF5 (Hayward et al., 2003). C1QTNF5 protein is composed of a C1Q-like globular head, a collagen-like domain and a signal peptide (Tu and Palczewski, 2012). The normal function of C1QTNF5 is currently unknown, however in the eye; it is thought to play a part in interactions between the RPE and the underlying basement membrane since the protein is secreted by the RPE and then attaches to the baso-lateral RPE plasma membrane (Mandal et al., 2006). Previous studies have indicated that mutant C1QTNF5 may affect cellular adhesion. Studies performed by Shu et al. (2006) found that HEK-EBNA cells stably expressing C1QTNF5 had reduced adhesion to laminin coated plates when compared with cells transfected with wild type protein. However, no differences were found when comparing fibronectin coated plates. These differences might have resulted from a number of causes including inherent adhesion differences in the cell lines (Kuschel et al., 2006). ECIS Medusa microarrays in this study were coated only with FCS and not with laminin, which may result in a different cell–substrate adhesion profile. Further studies will need to be performed using other substrates for comparison.

There have been previous studies on the effect of different agents on the adhesion and migration of RPE (Chan et al., 2010; Tsapara et al., 2010). Chan et al. have investigated the role of antioxidants on RPE adhesion and migration using ECIS wound healing assays. They studied the inhibitory effect of (-)-Epigallocatechin gallate (EGCG) (Chan et al., 2010), resveratrol (Chan et al., 2013) and lycopene (Chan et al., 2009) on platelet-derived growth factor (PDGF-BB) induced ARPE19 cell migration and adhesion to fibronectin. They reported that while all the three antioxidants have inhibited PDGF-BB induced RPE migration, only (-)-EGCG had an effect on adhesion to fibronection. Reservatrol and lycopene inhibited migration signaling pathways with no effect on adhesion. In vivo*,* optical coherence tomography studies in AMD suggested that aberrant adhesion/migration of intraretinal RPE might underlie progression to more advanced disease (Ho et al., 2011).

Finally, it is worth noting that RPE migration can be stimulated by an externally applied electrical field, and electrotaxis has been pointed out as a potential therapeutic strategy (Gamboa et al., 2010). However, RPE stimulated migration was observed at voltages orders of magnitude (50–300 mV) above the voltage used in this study (microvolts). The disease model-on-a-chip approach that have been developed in this study is well suited to investigate further the effect of different agents and drugs on migration and adhesion of both case and control cell lines, and can be adapted to the investigation of other inherited diseases.

Tissue-on-a-chip platforms are an emerging technology in drug discovery, tissue engineering and regenerative medicine (Borooah et al., 2013; Inoue and Yamanaka, 2011). So far, only a few studies restricted to the field of cardio-electrophysiology (Inoue and Yamanaka, 2011; Navarrete et al., 2013) have explored the combination of microelectrodes arrays and iPSC technologies. Human iPSCs-based models-on-a-chip show a new pathway for disease modeling and are beginning to establish a new paradigm for drug development and personalized medicine (Inoue and Yamanaka, 2011; Navarrete et al., 2013).

## 5 Conclusion

This study has demonstrated a reproducible and robust tissue-on-a-chip approach to quantitatively study a patient-specific retinal macular degeneration disease model. An hiPSC-RPE layer was directly established on ECIS microelectrodes where the platform enabled the label-free, real-time monitoring of hiPSC-RPE maturation in addition to injury and repair through the application of an integrated electrical wounding assay. This method mimicked RPE cell loss accompanying macular degeneration and was used to detect variations in migration rate between a cell line derived from a patient with late-onset retinal macular degeneration versus a control cell line derived from an unaffected siblings. This study points towards the role of cell adhesion in repair and will facilitate further studies to test the efficacy of potential therapeutic agents that modulate cell adhesion.

The tissue-on-a-chip AMD model is a powerful platform for translational studies. Combining hiPSCs technology with impedance sensing, it is amenable to a high throughput thus offering the opportunity to study patient-specific inherited macular degeneration in order to help achieve a better understanding of the disease mechanisms and identify potential therapies.
