# Supplementary figures and images for: Real-time quantitative monitoring of hiPSC-based model of macular degeneration on Electric Cell-substrate Impedance Sensing microelectrodes
Source: Biosens Bioelectron. 2015 Sep 15;71:445–55. doi: 10.1016/j.bios.2015.04.079 (PMC4456427; doi:10.1016/j.bios.2015.04.079)

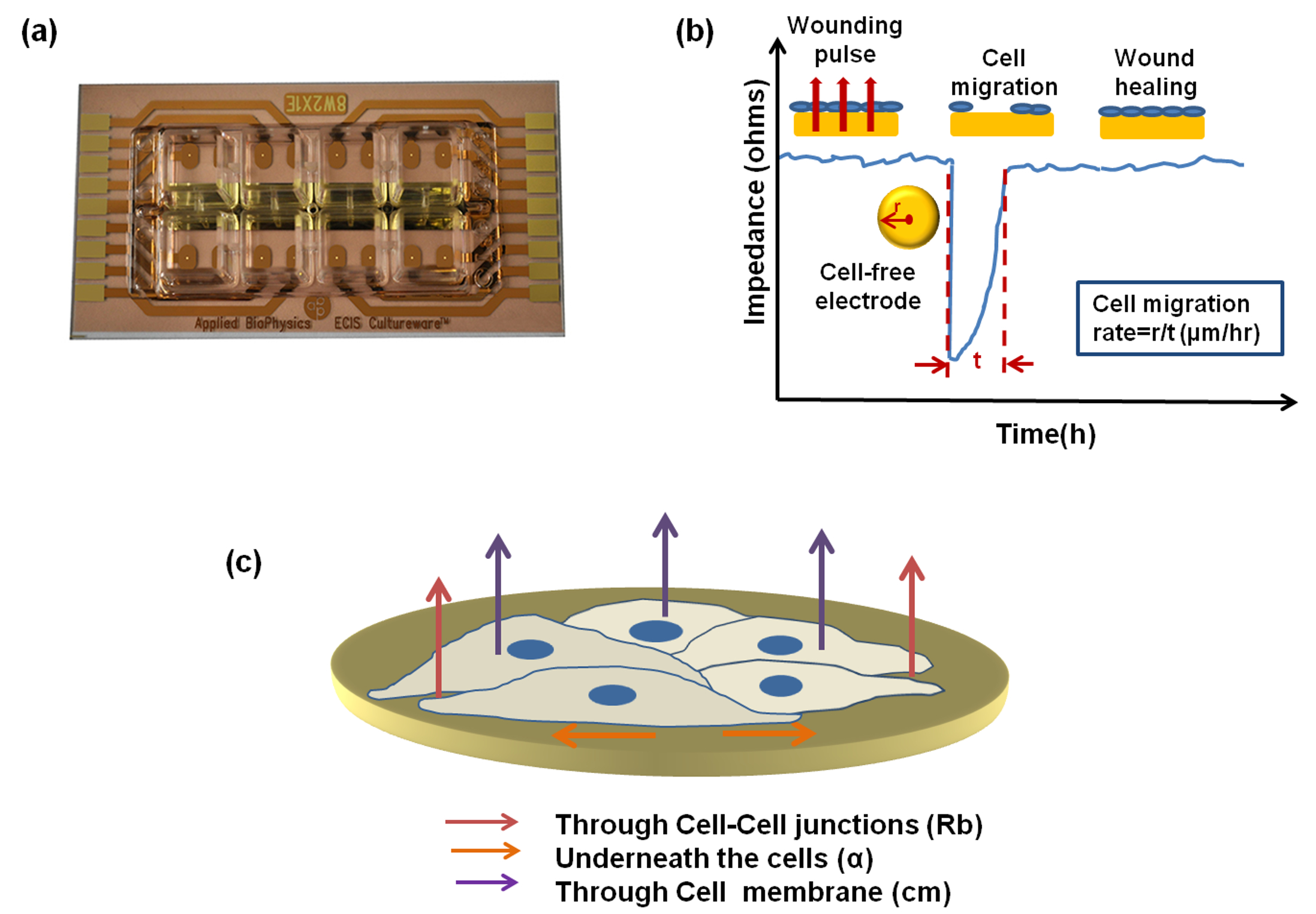

Supplement: Supplementary file 1 — Supplementary material [file mmc1.zip › mmc1.jpg]

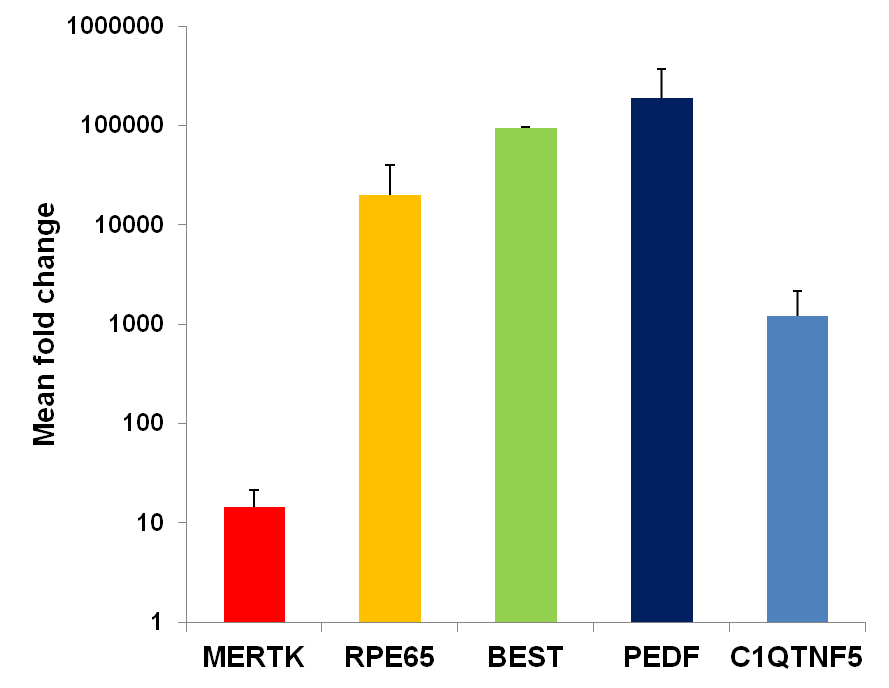

Supplement: Supplementary file 2 — Supplementary material [file mmc2.zip › mmc2.jpg]

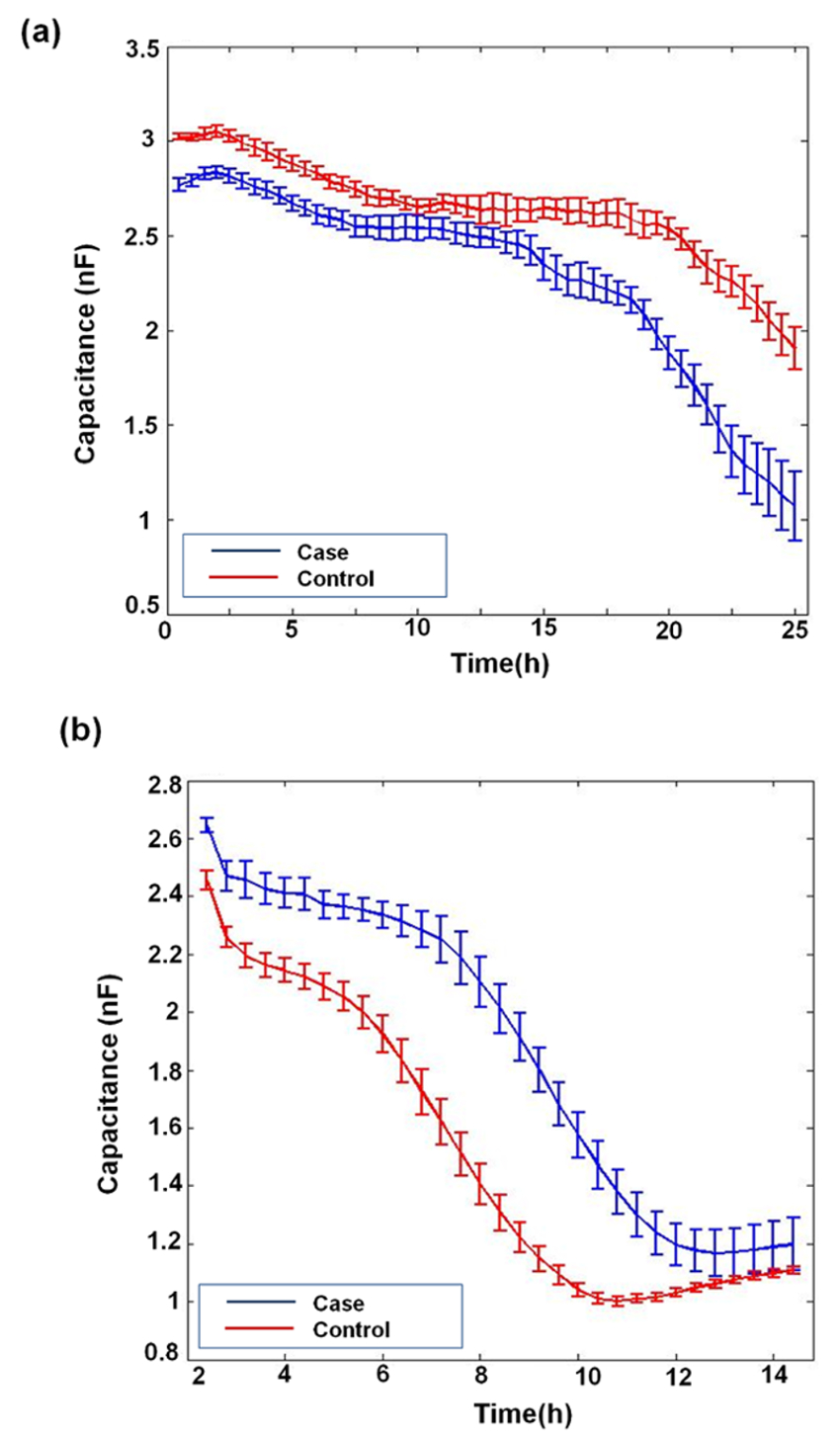

Supplement: Supplementary file 3 — Supplementary material [file mmc3.zip › mmc3.jpg]

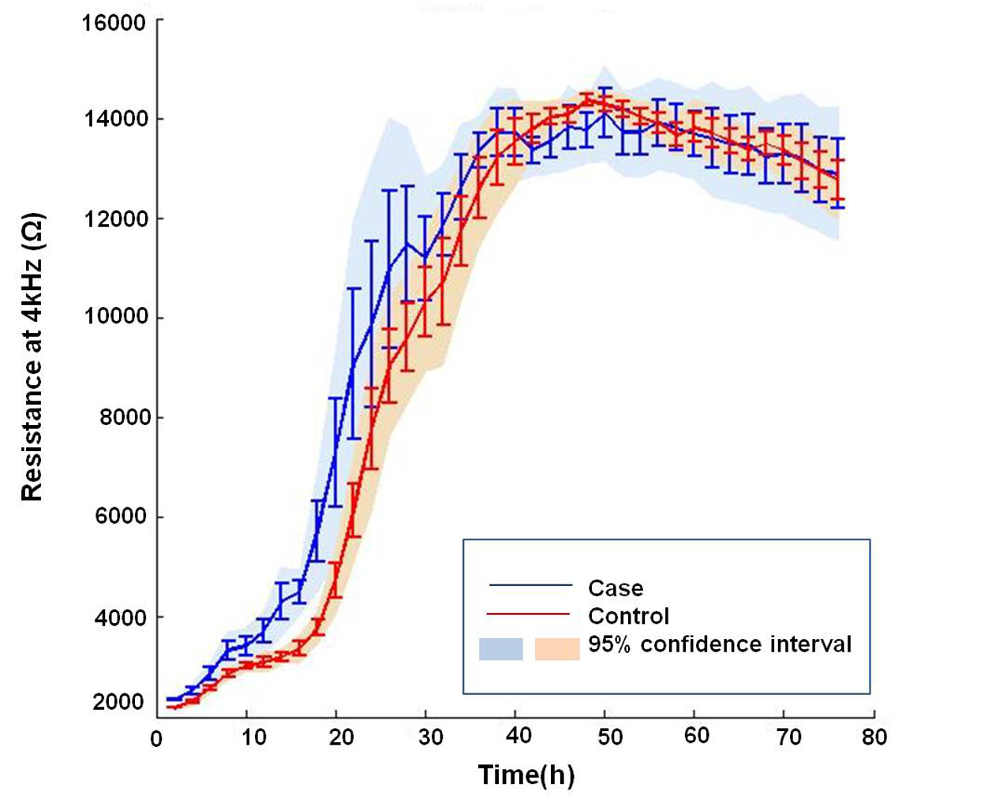

Supplement: Supplementary file 4 — Supplementary material [file mmc4.zip › mmc4.jpg]
