# Supplementary material for: Real-time quantitative monitoring of hiPSC-based model of macular degeneration on Electric Cell-substrate Impedance Sensing microelectrodes
Source: Biosens Bioelectron. 2015 Sep 15;71:445–55. doi: 10.1016/j.bios.2015.04.079 (PMC4456427; doi:10.1016/j.bios.2015.04.079)
Supplement: Supplementary file 5 — Supplementary material [file mmc5.pdf]

Supplementary Data

To

Real-time quantitative monitoring of hiPSCs-based model of macular degeneration on Electric Cell-substrate Impedance Sensing microelectrodes

W.Gamal[*2], S.Borooah[*1,4,5,6,7], S.Smith[2], I.Underwood[3],V.Srsen[2], S.Chandran[1,4,5,6,7], P.O.Bagnaninchi[+,1] and B. Dhillon[+7, 8]

1 MRC centre for regenerative medicine, The University of Edinburgh EH16 4UU,

2 Institute for Bioengineering, School of Engineering, The University of Edinburgh, EH9 3DW

3 Institute for Integrated Micro and Nano Systems, School of Engineering, The University of Edinburgh, EH9 3JF,

4 Centre for Clinical Brain Sciences, The University of Edinburgh, EH16 4SB, United Kingdom

5 Euan MacDonald Centre for MND Research, The University of Edinburgh, EH16 4SB, United Kingdom

6 Centre for Neuroregeneration, The University of Edinburgh, EH16 4SB, United Kingdom,

7 The Anne Rowling Regenerative Neurology Clinic, The University of Edinburgh, EH16 4SB, United Kingdom,

8 School of clinical sciences, The University of Edinburgh, EH16 4SB,

* Co-first authors, + co-senior authors

1. Supplementary Methods

Generation of hiPSC-RPE lines

HiPSC lines were derived from one patient with late-onset retinal macular degeneration (LORMD) and one unaffected sibling using previously established methods (Bilican B. et al., 2012 and Yusa K. et al., 2011). Briefly, fibroblasts were expanded from 3mm punch biopsies in medium containing 10 % fetal bovine serum (FBS), 89% DMEM and 1% penicillin/streptomycin. Once confluent cells were passaged to MEF feeder layers and reprogrammed using a Sendai reprogramming kit (Invitrogen). After four weeks, morphologically identifiable cells were selected and grown on MEF feeder layers in hiPSC medium containing DMEM/F12 (1:1), 20% KOSR, 1% MEM non-essential amino acids, 100 ng/ml bFGF, 1 mM L-glutamine and 0.1 mM β-mercaptoethanol. Long term culture was maintained feeder free in E8 (Life technologies) medium.

RPE differentiation was established using a variation of a previously published protocol (Meyer J.S. et al., 2009). Briefly, hiPSC colonies were lifted from MEF feeder layers with dispase (0.5 mg/ml) and collagenase (1 mg/ml) and grown as embryonic bodies (EB) for 4 days in EB medium containing DMEM/F12 (1:1), 20% KOSR, 1% MEM non-essential amino acids, 1 mM L-glutamine and 0.1 mM β-ME. At Day 5, EB medium was switched to neural induction medium (NIM) containing DMEM/F12 (1:1), 1% N2 supplement, MEM non-essential amino acids and 2 µg/ml heparin. At Day 7, suspended EB aggregates were plated to allow them to reattach to the culture plate, where they were grown for an additional 10 days in NIM. At Day 16, NIM medium was replaced with retinal differentiation medium (RDM) containing DMEM/F12 (3:1), 2% B27 supplement (without retinoic acid), MEM non-essential amino acids and penicillin/streptomycin. The cells were maintained as adherent cultures in RDM until the appearance of pigmented RPE cells. Large patches of pigmented RPE cells were micro-dissected and then grown on laminin coated plates initially in 10% FBS/RDM

90% for two days, followed by 2% FBS/98% RDM till confluent before switching to RDM. RPE validation was performed using RT-PCR and immunostaining.

Biochemical Adhesion Assay

Single cell suspensions of hiPSC-RPE were prepared by enzymatic dissociation with 0.1% trypsin-EDTA for 10 minutes. The concentration was resuspended into 1.0 x 10^6 cells/ml in assay buffer. 100 μL of the cell suspension was added to each well of an extra cellular matrix adhesion kit (Millipore) before incubation for 1-2 hrs at 37°C in a CO_2 incubator. After incubation media was discarded and the wells were washed gently 3 times with 200 μL assay Buffer. 100 μL of cell stain solution was added to each well and were incubated for 5 minutes at room temperature. Stain was gently removed and the wells were washed 5 times with deionized water before air drying. 100 μL of extraction buffer was added to each well and the wells were incubated with shaking in an orbital shaker for 5 minutes. The absorbance of the wells were analysed at 545 nm on a microplate reader.

Quantitative data analysis

Sigmoid fitting: The healing graphs were fitted to a sigmoid curve in which the hill slope and inflection points were calculated using the following equation for a deeper insight into healing kinetics.

Equation (1):

$$\boldsymbol{y = a + \frac{b-a}{1+10^{(c-x)d}}}$$

Where; a is the y value at the sigmoid bottom plateau, b is the y value at the sigmoid top plateau, c is the x value when the response is half way between top and bottom and d is the hill slope or slope factor and has no units.

Average cell migration: The migration rate of the case and control cell lines after electrical wounding was determined according to the equation:

Equation (2): $m = r/t$ (μm/hr)

Where; m is the migration rate, r is the electrode's radius (125 μm) and t is the time required for the impedance to reach its values prior to wounding.

2. Supplementary Figures

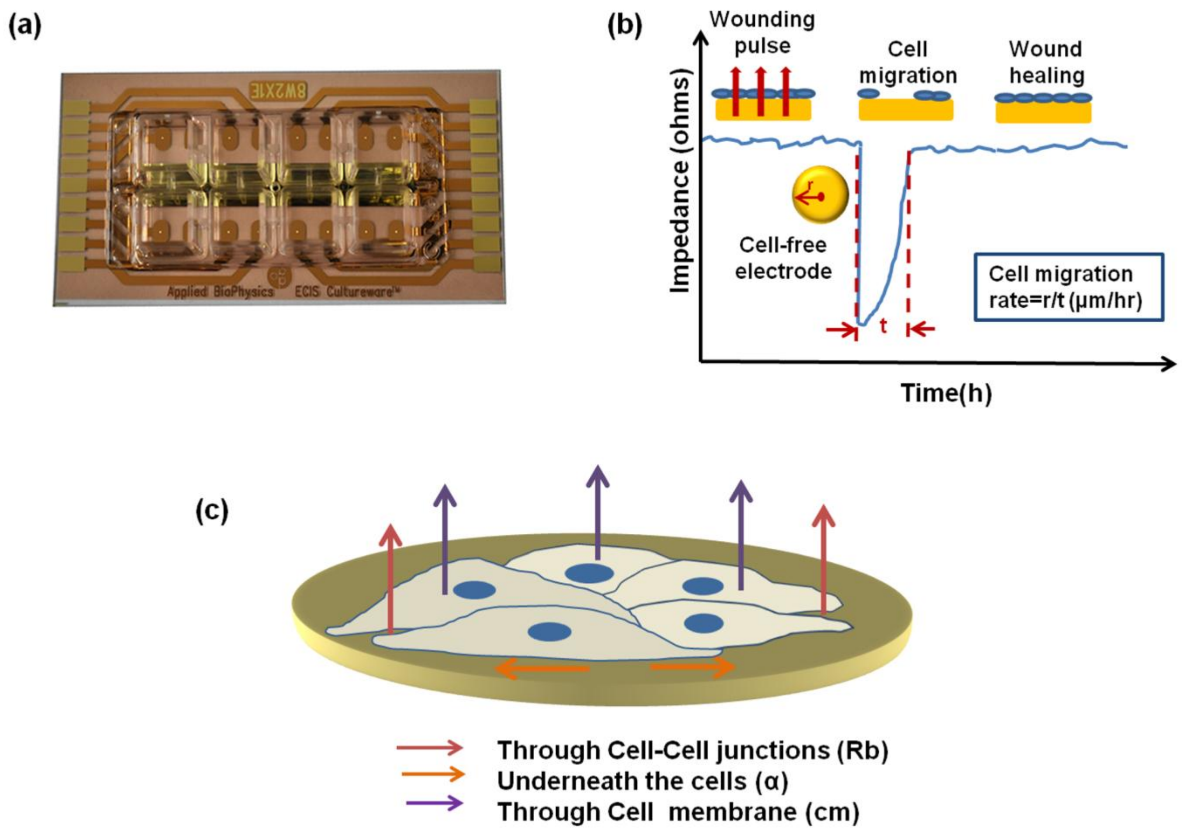

Supplementary Figure 1: Electric Cell-Substrate Impedance Sensing. (a) ECIS medusa arrays having 8 wells of 0.8 cm^2 surface area with two 250 μm gold microelectrodes per well. All cell lines were cultured using this type of arrays. Each microelectrode was normally covered by 50-100 cells of the confluent cell layer. (b) Electrical wound healing assay: in its wounding mode, ECIS apply an elevated current pulse to kill the cells on top of the microelectrode and creating a reproducible 250 μm circular wound through irreversible electroporation. This is mirrored by a drop in impedance to that of the cell-free electrode level followed by a gradual increase reflecting cell migration to repopulate the microelectrodes until a plateau is reached indicating full recovery. (c) ECIS model: the built-in ECIS model scans impedance measurements through various frequencies to recognize the current pathways and translates it into data on cell-cell junctions, cell-electrode adhesion

as well as the cell membrane capacitance. This is based on the fact that at low frequencies, the current flows underneath and in between the cells reflecting how tight the cell-cell junctions are and how well-adhered are the cells to the electrodes. At high frequencies, the current can capacitively couple through the cells revealing data on the intracellular properties and the integrity of the cell plasma membrane. We used this model to study the cell adhesion parameter (α) to investigate between case and control adhesion properties.

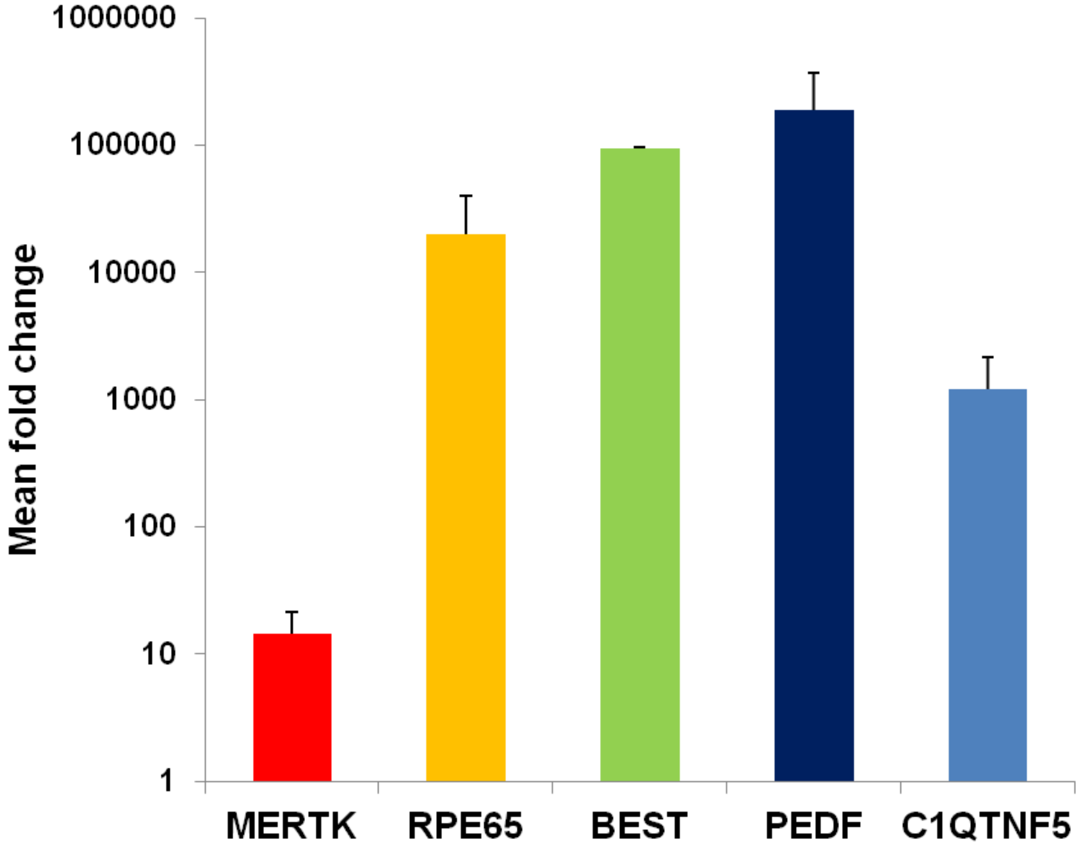

Supplementary Figure 2 Expression of RPE genes and proteins in hiPSC-RPE. qPCR (40 cycles) showing the relative fold change of expression of the mRNA of RPE monolayers relative to hiPSC demonstrating maturation of RPE (data from all lines were pooled).

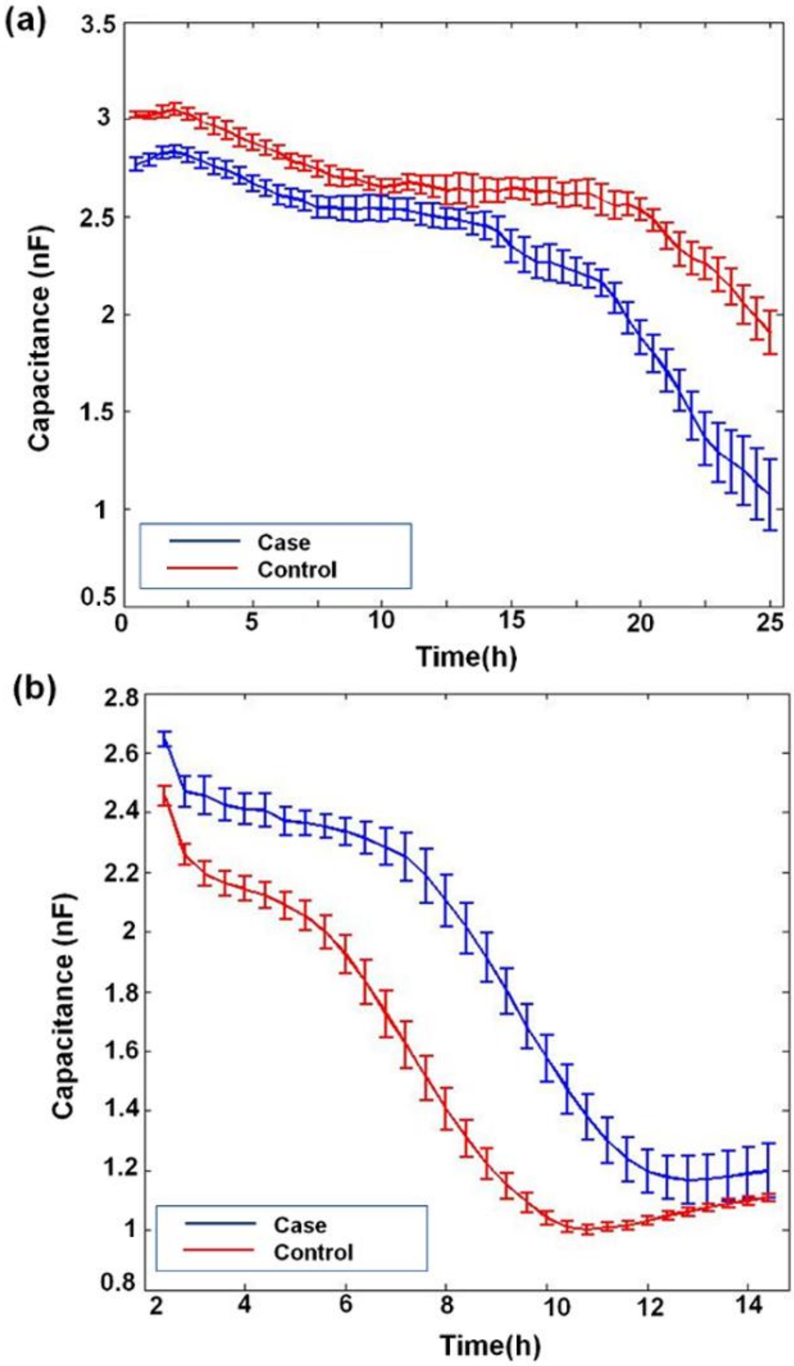

Supplementary Figure 3: Capacitance measurements. (a) Attachment and spreading phase: The capacitance is the out-of-phase component of the impedance measurements. While the measured resistances increase with cells attaching and spreading onto the electrodes, the capacitance measurements decrease with cell coverage due to the cells insulating effect. During the attachment phase, the capacitance of the case cell line decreased faster than that of the control cell line suggesting that they have a stronger adhesion to the electrode surface. (b) Wound healing: When cells are wounded, the

capacitance increases ideally to the value of the cell-free electrode and starts decreasing again with healing to reach the pre-wounding values. The capacitance measurements of the control cell lines showed a faster decrease reaching a plateau before the case cell line. This was attributed to the control cell line having a higher migration rate than the case cell line.

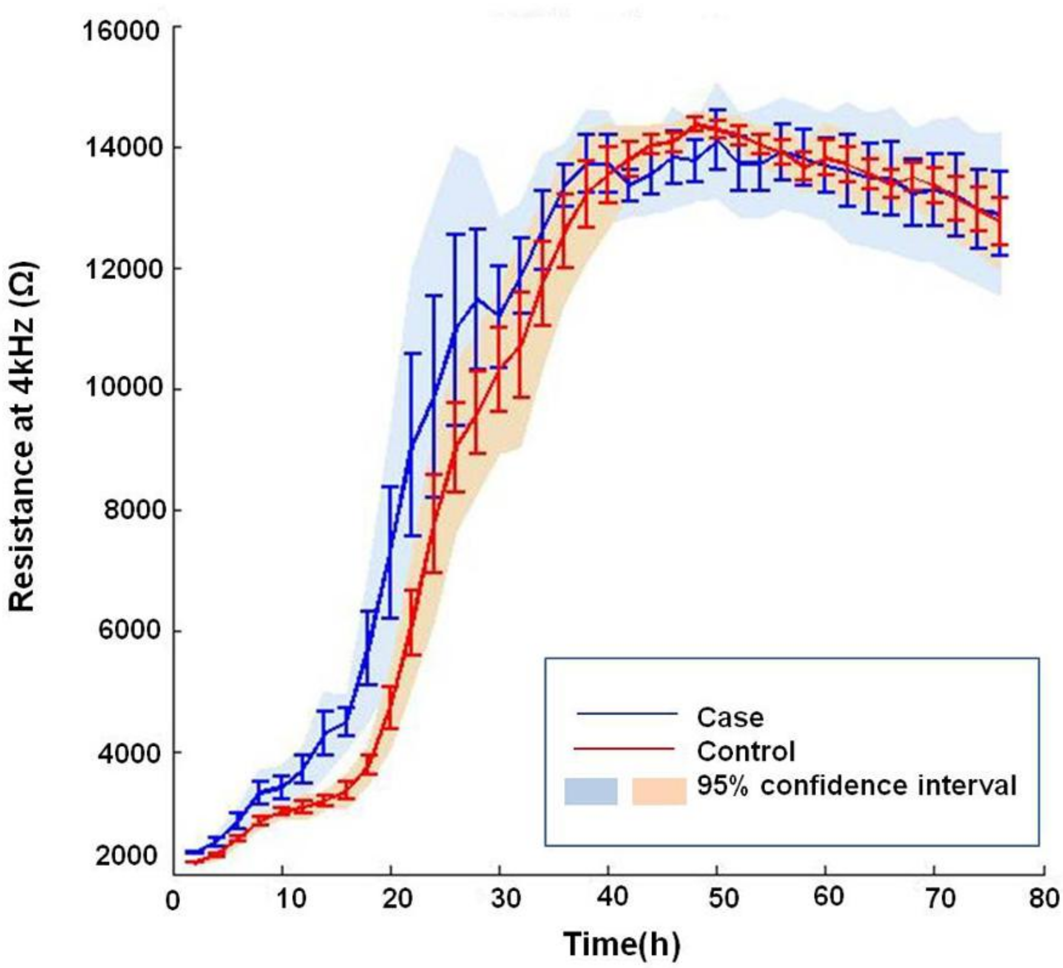

Supplementary Figure 4: Differentiation and maturation kinetics. During the attachment phase (first 24h), the case cell line adhered to the microelectrodes faster than the control cell line expressing a higher resistance and reaching a plateau first. After this phase however, both the case and control cell lines followed a similar trend during differentiation and maturation.
